# Quality of life changes over time and predictors in a large head and neck patients’ cohort: secondary analysis from an Italian multi-center longitudinal, prospective, observational study—a study of the Italian Association of Radiotherapy and Clinical Oncology (AIRO) head and neck working group

**DOI:** 10.1007/s00520-023-07661-2

**Published:** 2023-03-17

**Authors:** Anna Viganò, Francesca De Felice, Nicola Alessandro Iacovelli, Daniela Alterio, Rossana Ingargiola, Alessia Casbarra, Nadia Facchinetti, Olga Oneta, Almalina Bacigalupo, Elena Tornari, Stefano Ursino, Fabiola Paiar, Orietta Caspiani, Alessia Di Rito, Daniela Musio, Paolo Bossi, Patrizia Steca, Barbara Alicja Jereczek-Fossa, Letizia Caso, Nicola Palena, Andrea Greco, Ester Orlandi

**Affiliations:** 1 Department of Human and Social Sciences, University of Bergamo, Bergamo, Italy; 2 Department of Radiotherapy, Policlinico Umberto I “Sapienza” University of Rome, Rome, Italy; 3 Radiotherapy 2 Unit, Fondazione IRCCS Istituto Nazionale Dei Tumori Di Milano, Milan, Italy; 4 Division of Radiation Oncology, IEO, European Institute of Oncology IRCCS, Milan, Italy; 5 Department of Oncology and Hemato-Oncology, University of Milan, Milan, Italy; 6 Radiation Oncology Policlinico San Martino IRCCS, Genoa, Italy; 7 Department of Radiation Oncology, S. Chiara University Hospital, Pisa, Italy; 8 Radiation Oncology Department, Ospedale “S. Giovanni Calibita” Fatebenefratelli, Rome, Italy; 9 IRCCS Istituto Tumori “Giovanni Paolo II”, Bari, Italy; 10 Medical Oncology, Department of Medical and Surgical Specialties, Radiological Sciences and Public Health University of Brescia, ASST-Spedali Civili, Brescia, Italy; 11 Department of Psychology, University of Milan “Bicocca”, Milan, Italy; 12 Department of Human Sciences, Libera Università Maria SS. Assunta University, Rome, Italy

**Keywords:** Head and neck cancer, MDASI-HN, Quality of life, Longitudinal trajectories, HPV, Radiotherapy

## Abstract

**Purpose:**

The present study examined the longitudinal trajectories, through hierarchical modeling, of quality of life among patients with head and neck cancer, specifically symptoms burden, during radiotherapy, and in the follow-up period (1, 3, 6, and 12 months after completion of radiotherapy), through the M.D. Anderson Symptom Inventory Head and Neck questionnaire, formed by three factors. Furthermore, analyses were conducted controlling for socio-demographic as well as clinical characteristics.

**Methods:**

Multi-level mixed-effects linear regression was used to estimate the association between quality of life and time, age, gender, household, educational level, employment status, ECOG performance status, human papilloma virus (HPV) status, surgery, chemotherapy, alcohol intake, and smoking.

**Results:**

Among the 166 participants, time resulted to be a predictor of all the three questionnaire factors, namely, general and specific related symptoms and interference with daily life. Moreover, regarding symptom interference with daily activities factor, HPV-positive status played a significant role. Considering only HPV-negative patients, only time predicted patients’ quality of life. Differently, among HPV-positive patients, other variables, such as gender, educational level, alcohol use, surgery, age at diagnosis, employment status, and ECOG status, resulted significant.

**Conclusion:**

It was evident that quality of life of patients with head and neck cancer declined during RT, whereas it slowly improved after ending treatment. Our results clarified the role of some socio-demographic and clinical variables, for instance, HPV, which would allow to develop treatments tailored to each patient.

## Introduction

Head and neck carcinoma (HNC) is becoming common worldwide, and it is anticipated to rise by 30% accounting for an estimated 1.08 million new cancer cases annually by 2030 [1]. In particular, the increasing rates of human papilloma virus (HPV)-related tumors, with better prognosis compared to the counterpart, have contributed to this high prevalence of HNC especially in the United States of America and Western Europe [2, 3]. Currently, regardless of HPV status, evidenced-based treatments are multimodal [4] and may produce several physical complications and psychological distress, which may persist beyond treatment [5, 6]. The main treatment-related side effects are oral mucositis, taste impairment, salivary gland dysfunction, xerostomia, incapacity to chew and swallow, bacterial and fungal infections, neuropathy, trismus, and skin changes and reactions of the treated area [5, 7–11]. All these complications impair patients’ ability to perform on daily activities [10], resulting in social withdrawal, mental, and emotional distress [12] and impacting patients’ health-related (HR) quality of life (QoL) domains but also more general QoL domains [13, 14]. HRQoL may be described as a subjective and multi-dimensional concept related to one’s perception of well-being and satisfaction with one’s own health as well daily life functioning [13], which encompasses physical, psychological, and social functioning and disease-treatment related symptoms and side effects [15]. Thus, it may be considered a subset of the broader concept of QoL, defined as “an individual’s perception of their position in life in the context of the culture and value systems in which they live and in relation to their goals, expectations, standards and concerns” [16]. Accordingly, we have decided to focus on the more comprehensive term of QoL.

As it was abovementioned said, HNC patients’ face unique physical, emotional, and psychological challenges and life disruptions, in comparison to other cancer sites [17]. Hence, understanding QoL changes and patients’ needs [18] during and after therapy is essential to manage the disease more effectively and to set up rehabilitative strategies for the patients [19]. Longitudinal studies reported that QoL usually decreases during radiation therapy (RT) and starts to improve 3–6 months after treatment, with a global amelioration one year after RT end, without a complete return to pre-treatment status, and with a pattern varies depending on the dimension of QoL evaluated [20–24].

In addition, information about clinical and treatment-related predictors impacting on improvement and recovery on QOL is not comprehensive enough so far. A multi-center longitudinal, prospective, observational study of consecutive HNC patients, treated at seven Italian Oncology Radiotherapy Departments, was conducted on behalf of the Italian Association of Radiotherapy and Clinical Oncology (AIRO) Head and Neck Working Group. The first endpoint was the Italian language psychometric validation of the M.D. Anderson Symptom Inventory Head and Neck (MDASI-HN) questionnaire [25]. Here, we present results of secondary endpoints: (i) investigate QoL in patients with HNC using the MDASI-HN module to measure symptom burden during RT and in the follow-up period, namely, (1, 3, 6, and 12 months after completion of RT) and (ii) analyze whether QoL may be predicted by socio-demographic and clinical characteristics.

## Method

### Procedure

This was a multi-center prospective longitudinal observational study of consecutive HNC patients treated with RT at seven Italian Oncology Radiotherapy Departments, from 2016 to 2019. Eligibility criteria were patients with a squamous cell carcinoma of the head and neck (including oral cavity, oropharynx, larynx, and hypopharynx); age ≥ 18 years old; Eastern Cooperative Oncology Group (ECOG) performance status < 2; and good knowledge of Italian language. Exclusion criteria included history of cognitive or psychiatric disorders, synchronous tumors, or previous RT to the head and neck region. Treatment details were previously described [25]. Briefly, all patients were treated with (chemo)radiotherapy ((C)RT) with definitive or adjuvant intent (postoperative), based on primary and disease stage. If needed, type of surgical approach and induction chemotherapy regimen were chosen by the respective professionals.

The study was approved by the Ethical Committee of Fondazione IRCCS Istituto Nazionale dei Tumori in Milan (prot. INT 29/15). All patients signed study-specific informed consent and answered to the questionnaire after the physician visit.

Questionnaire measure and socio-demographic and clinical variables were collected at different time points: pre-treatment (before RT); weekly during RT (6–7 weeks); and in the follow-up period, specifically 1, 3, 6, and 12 months after RT.

### Questionnaire and data collection

The MDASI-HN is a brief and reliable patient-reported outcome measure (PROM) questionnaire developed to investigate symptoms severity, specifically general cancer-related symptoms (GC-RS), head and neck cancer-related symptoms (HNC-RS), and symptoms interference with daily activities (SIDA) [26, 27]. It contains 13 items representing the most common symptoms among all cancer types (such as fatigue level, lack of appetite and vomiting) and 9 items specific to HNC (such as problems with tasting food, choking or coughing and difficulty swallowing or chewing). These items assess the presence and severity of symptoms during the previous 24 h, rating them on a 11-point scale from “not present” (0) to “as bad as you can imagine” (10). The last 6 items concern how these symptoms interfere with daily activities, including work, walk, and relationship with other; these assess how general and specific cancer symptoms interfere with patients’ activities during the past 24 h. These items are rated on a scale ranging from “do not interfere” (0) to “interfered completely” (10) [27].

Clinical and socio-demographic characteristics, including age, sex, living situation, educational level, employment status, alcohol consumption and tobacco use, ECOG performance status, human papillomavirus (HPV) status, RT setting (adjuvant vs. definitive), and concomitant systemic therapy, were also collected.

### Statistical analysis

Data were analyzed using IBM SPSS Statistics version 25 (IBM, Armonk, NY, USA).

Multi-level mixed-effects linear regression estimated the association between QoL and time as well as with clinical and socio-demographic variables. We opted for such a hierarchical approach as it (a) permits to model random effects (intercepts and slopes) of time and (b) permits to treat variables as nested within other variables; in particular, for the present study, the various timepoints are nested under each participant. We also investigated the missing and response rate at each timepoint as percentage (e.g., number of participants who responded at week x/total number of participants*100). The following variables were investigated: time (in weeks), age, sex, living situation, educational level, employment status, alcohol consumption and tobacco use, ECOG performance status, HPV status, RT setting, and concomitant systemic therapy. Last, we set alpha at *p* < 0.05.

## Results

### Participants

From January 2016 to December 2019, 166 HNC patients were enrolled and received (C)RT. The response rate at the beginning of the study was high in all the three dimensions, and at time 1, it ranged from 95.78% (GC-RS) to 93.37% (SIDA); however, it slowly decreased from the last week of treatment. Indeed, the missing rate gradually increased in the follow-up period. At week 8, missing rate was of 31.93% for all three factors of the MDASI-HN, whereas it raised at 60.84% at week 52. Patient socio-demographic characteristics are shown in Table 1, while tumor and treatment characteristics are shown in Table 2. Most of the patients, specifically 79%, had locally advanced disease according to TNM 7th edition.

**Table 1:** Patients’ socio-demographic characteristics

Characteristics	Frequencies	(%)
Gender		
Male	119	71.7
Female	47	28.3
Age (years), mean (SD) = 61.69 (11.01); range = 24–93		
Living situation		
Alone	15	9.0
With someone	129	77.7
Missing	22	13.3
Educational level		
Lower school	52	31.3
Higher school	92	55.4
Missing	22	13.3
Employment status		
Employed	58	34.9
Unemployed	85	51.2
Missing	23	13.9

**Table 2:** Patients’ clinical characteristics

Characteristics	Frequencies	(%)
Tumor site		
Hypopharynx	8	4.8
Larynx	29	17.5
Oral cavity	34	20.5
Oropharynx	91	54.8
Missing	4	2.4
Stage disease (according to TNM 7th edition)		
I	5	3
II	14	8.4
III	22	13.3
IV	109	65.7
Missing	16	9.6
Performance status*		
0	105	63.3
1	58	34.9
Missing	3	1.8
Smoker		
Current/former	111	66.9
Never	47	28.3
Missing	8	4.8
Alcohol consumption		
Current/former	57	34.3
Never	55	33.1
Missing	54	32.5
HPV status		
Negative	100	60.2
Positive	66	39.8
Radiation therapy intent		
Adjuvant (45 Gy–66 Gy) with SyT	20	12.0
Adjuvant (45 Gy–66 Gy) without SyT	32	19.3
Definitive (66 Gy–72 Gy) with SyT	84	50.6
Definitive (66 Gy–72 Gy) without SyT	28	16.9
Missing	2	1.2

*According to Eastern Cooperative Oncology Group (ECOG). *HPV*, human papilloma virus; *Gy*, gray; *SyT*, systemic therapy

### Socio-demographic and clinical and variables and changes of QoL over time

Considering the whole sample, first, hierarchical linear model analysis was conducted on the factor GC-RS as the dependent variable in a stepwise fashion and indicated that the best model was the one including the linear, quadratic, and cubic effects of time, and both the intercepts and the slope of time (linear) as random effects. Subsequently, the other variables were also entered in the analyses. After entering them, the random effect of the slope was no longer significant and was hence excluded. Table 3 shows results of this model.

**Table 3:** Hierarchical linear model analysis conducted with the whole sample (*n* = 166)

Independent variables	GC-RS factor	HNC-RS factor	SIDA factor
	*b* (95% CI)	*b* (95% CI)	*b* (95% CI)
Gender	-0.45 (-1.18; 0.27)^ns^	-0.45 (-1.26; 0.37)^ns^	-0.67 (-1.67; 0.33)^ns^
Age	0.0001 (-0.03; 0.03)^ns^	-0.01 (-0.05; 0–02)^ns^	0.005 (-0.03; 0.04)^ns^
Living situation	0.24 (-0.87; 1.35)^ns^	0.20 (-1.04; 1.44)^ns^	0.58 (-0.95; 2.10)^ns^
Educational level	0.10 (-0.57; 0.77)^ns^	0.46 (-0.28; 1.21)^ns^	0.16 (-1.07; 0.76)^ns^
Employment status	-0.33 (-1.08; 0.4)^ns^	-0.28 (-1.11; 0.55)^ns^	0.60 (-0.42; 1.61)^ns^
ECOG	0.19 (-0.48; 0.87)^ns^	0.32 (-0.43; 1.07)^ns^	0.21 (-0.71; 1.13)^ns^
Smoker	0.51 (-0.21; 1.23)^ns^	0.34 (-0.46; 1.14)^ns^	0.48 (-0.05; 1.47)^ns^
Alcohol use	-0.51 (-1.18; 0.16)^ns^	-0.16 (-0.91; 0–59)^ns^	-0.68 (-1.59; 0.24)^ns^
HPV	-0.59 (-1.32; 0.15)^ns^	-0.54 (-1.36; 0.28)^ns^	-1.59 (-2.60; -0.59)^**^
Surgery	-0.70 (-0.79; 0.65)^ns^	-0.49 (-1.3; 0.32)^ns^	-0.11 (-1–10; 0.89)^ns^
Chemotherapy	0.34 (-0.35; 1.05)^ns^	0.38 (-0.4; 1.16)^ns^	0.70 (-0.26; 1.66)^ns^
Time			
Linear effect	0.33 (0.27; 0.38)^***^	0.54 (0.46; 0.62)^***^	0.37 (0.29; 0.45)^***^
Quadratic effect	-0.02 (-0.02; -0.01)^***^	-0.03 (-0.03; -0.02)^***^	-0.02 (-0.02; -0.01)^***^
Cubic effect	0.0002 (0.0001; 0.0002)^***^	0.0003 (0.0002; 0.0003)^***^	0.0002 (0.0001; 0.0002)^***^

*GC-RS*, general cancer-related symptoms; *HNC-RS*, head and neck cancer-related symptoms; *SIDA*, symptom interference with daily activities; *ECOG*, Eastern Cooperative Oncology Group; *HPV*, human papilloma virus; *CI*, confidence interval; *ns*, not significant; ^*^p < 0.05, ^**^p < 0.01, and ^***^p < 0.001 (two-tailed)

A second analysis was conducted on the factor HNC-RS as the dependent variable in the same stepwise fashion as for the first dimension. The analyses showed that the best fitting model included the linear, quadratic, and cubic trend and the random effect of the intercepts (linear). Subsequently, the other variables were entered in the analyses. None of the variables considered reached significance except for time (Table 3).

A third analysis was conducted on SIDA as the dependent variable, again in a stepwise fashion. The analyses showed that the best fitting model included the three effects of time (linear, quadratic, and cubic) and the random effects of the intercepts and the slope (linear). As for the first factor, once the other variables were entered in the analyses, the random effect of the slope was no longer significant; hence, it was excluded. The HPV status and the linear, quadratic, and cubic effects of time were significant (Table 3).

**Fig. 1:**
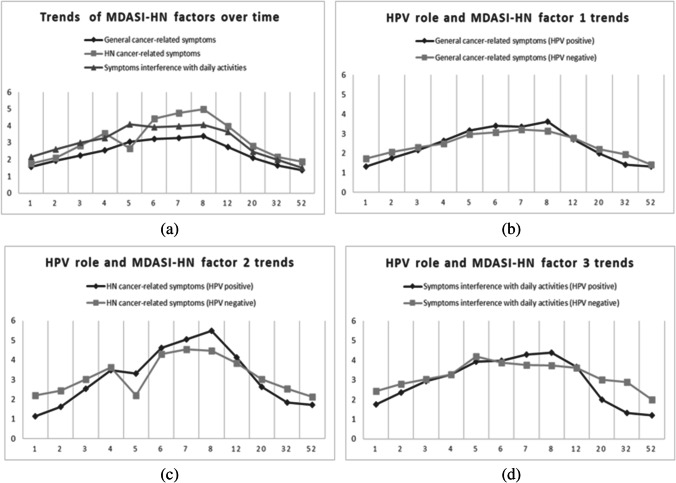
Longitudinal trajectories of MDASI-HN. The graph shows the longitudinal trajectories of MDASI-HN. The time is represented on the horizontal axis in weeks. The MDASI-HN score is represented on the vertical axis, but it does not replicate the MDASI-HN response scale from 0 to 10. (**a**) Trends of MDASI-HN factors; (**b**) trends of MDASI-HN general cancer-related symptoms (GC-RS) factor, distinguishing between HPV-positive patients and HPV-negative patients; (**c**) trends of MDASI-HN HNC-RS factor, distinguishing between HPV-positive patients and HPV-negative patients; (**d**) trends of MDASI-HN SIDA factor, distinguishing between HPV-positive patients and HPV-negative patients

As Fig. 1a shows, for all three MDASI factors, there was a trend whereby the scores increased from week 1 to week 8 (with some fluctuation between week 4 and week 8), followed by a decrease from week 8 to week 52. Considering that a higher score indicates lower QoL, the results indicated a worsening in the first eight weeks, followed by a slow return to a better QoL.

### Changes of QoL over time: the role of HPV

Since the amount of patient diagnosed with oropharynx cancer outnumbered those with other tumor locations, the same analyses as above were conducted only for those cases where the location of the tumor was the oropharynx, considering patients HPV positive and negative separately.

In relation to HPV-negative patients, as can be seen in Table 4, for the GC-RS factor, the best fitting model included linear, quadratic, and cubic trend of time; all the other variables; and the random effect of the intercepts (linear). This model showed that the linear, quadratic, and the cubic effects of time were all significant. For the HNC-RS factor, the best model was the one including the fixed effect of linear, quadratic, and cubic effects of time and that of all the other variables, plus intercepts of time (linear) as random effect. Again, linear, quadratic, and cubic effects of time were all significant. The analysis conducted on the SIDA factor showed that the best model was the one including the three effects of time (linear, quadratic, and cubic), all the other variables, and the random effects of the intercepts (linear). The model showed that the linear, quadratic, and cubic effects of time were all significant. In all these three dimensions, none of the other variables considered reached significance.

**Table 4:** Hierarchical linear model analysis conducted with the oropharynx HPV-negative patients (*n* = 100)

Independent variables	GC-RS factor	HNC-RS factor	SIDA factor
	*b* (95% CI)	*b* (95% CI)	*b* (95% CI)
Gender	-1.21 (-2.76; 0.33)^ns^	-1.39 (-2.96; 0.17)^ns^	-1.43 (-3.73; 0.88)^ns^
Age	0.03 (-0.10; 0.15)^ns^	0.07 (-0.05; 0.20)^ns^	0.09 (-009; 0.28)^ns^
Educational level	-0.35 (-1.95; 1.26)^ns^	0.28 (-1.34; 1.90)^ns^	-0.68 (-3.08; 1.72)^ns^
Employment status	-0.55 (-3.98; 2.89)^ns^	0.41 (-3.07; 3.89)^ns^	1.17 (-3.96; 6.31)^ns^
ECOG	-0.36 (-1.96; 1.24)^ns^	-0.46 (-2.07; 1.16)^ns^	-0.16 (-2.55; 2.23)^ns^
Smoker	0.01 (-1.88; 1.90)^ns^	-0.93 (-2.83; 0.97)^ns^	-0.26 (-3.07; 2.55)^ns^
Alcohol use	-0.30 (-2.58; 1.98)^ns^	1.36 (-0.94; 3.66)^ns^	0.74 (-2.66; 4.14)^ns^
Surgery	0.07 (-2.28; 2.43)^ns^	0.04 (-2.34; 2.41)^ns^	0.91 (-2.60; 4.43)^ns^
Chemotherapy	0.87 (-1.75; 3.49)^ns^	0.28 (-2.36; 2.92)^ns^	0.61 (-3.30; 4.52)^ns^
Time			
Linear effect	0.35 (0.22; 0.49)^***^	0.58 (0.39; 0.77)^***^	0.51 (0.32; 0.70)^***^
Quadratic effect	-0.02 (-0.03; -0.01)^***^	-0.03 (-0.04; -0.02)^***^	-0.03 (-0.04; -0.02)^***^
Cubic effect	0.0003(0.0002; 0.0004)^***^	0.0004 (0.0002; 0.0005)^***^	0.0004 (0.0002; 0.0005)^***^

*GC-RS*, general cancer-related symptoms; *HNC-RS*, head and neck cancer-related symptoms; *SIDA*, symptom interference with daily activities; *ECOG*, Eastern Cooperative Oncology Group; *HPV*, human papilloma virus; *CI*, confidence interval; *ns*, not significant; ^*^p < 0.05, ^**^p < 0.01, and ^***^p < 0.001 (two-tailed)

In relation to HPV-positive patients (Table 5), for the first factor, the best model was the one including the fixed effect of linear, quadratic, and cubic effects and that of all the other variables, plus intercepts of time (linear) as random effect. The model showed that the linear, quadratic, and the cubic effects of time were all significant. Further, the effect of gender, age at diagnosis, educational level, surgery, and alcohol use were also significant. The estimated marginal means indicated that male patients (*M* = 2.16, SE = 0.42), with a higher educational level (*M* = 2.11, SE = 0.33), who had surgery (*M* = 2.15, SE = 0.53), and those who use alcohol (*M* = 2.22, SE = 0.38) had lower scores than females (*M* = 3.30, SE = 0.37), who had a low educational level (*M* = 3.35, SE = 0.45), who had not the surgery done (*M* = 3.31, SE = 0.32), and who never drink alcohol (*M* = 3.24, SE = 0.40). For the second factor, the best fitting model included linear, quadratic, and cubic trend of time; all the other variables; and the random effect of the intercepts (linear). The model showed that the linear, quadratic, and the cubic effects of time were all significant. The effect of educational level and ECOG status was also significant. Patients with a lower educational level (*M* = 5.38, SE = 0.47) and those fully active (ECOG 0) (*M* = 4.93, SE = 0.41) showed higher scores than those with higher educational level (*M* = 3.56, SE = 0.35) and restricted in physically strenuous activity (ECOG 1) (*M* = 4.01, SE = 0.43). For the third factor, the best model was the one including the fixed effect of linear, quadratic, and cubic effects of time and that of all the other variables, plus intercepts of time (linear) as random effect. Again, the linear, quadratic, and the cubic effects of time were all significant. The effects of gender, age at diagnosis, employment status, and alcohol use were also significant. Patients who were female (*M* = 3.70, SE = 0.62), employed (*M* = 3.76, SE = 0.68), and never use alcohol (*M* = 3.57, SE = 0.66) showed higher scores that males (*M* = 2.08, SE = 0.70), unemployed (*M* = 2.02, SE = 0.63), and alcohol user (*M* = 2.21, SE = 0.63).

**Table 5:** Hierarchical linear model analysis conducted with the oropharynx HPV-positive patients (*n* = 66)

Independent variables	GC-RS factor	HNC-RS factor	SIDA factor
	*b* (95% CI)	*b* (95% CI)	*b* (95% CI)
Gender	-1.14 (-1.88; -0.41)^**^	-0.72 (-1.49; 0.04)^ns^	-1.62 (-2.85; -0.39)^*^
Age at diagnosis	0.04 (0.003; 0.08)^*^	0.03 (-0.02; 0.07)^ns^	0.11 (0.04; 0.18)^**^
Living situation	-0.58 (-1.42; 0.26)^ns^	-0.36 (-1.23; 0.51)^ns^	-0.74 (-2.14; 0.65)^ns^
Educational level	1.24 (0.48; 1.99)^**^	1.82 (1.05; 2.60)^***^	0.50 (-0.75; 1.75)^ns^
Employment status	0.31 (-0.43; 1.04)^ns^	0.39 (-0.37; 1.15)^ns^	1.74 (0.52; 2.96)^**^
ECOG	0.14 (-0.66; 0.94)^ns^	0.91 (0.09; 1.73)^*^	0.54 (-0.79; 1.88)^ns^
Smoker	0.62 (-0.11; 1.36)^ns^	0.54 (-0.22; 1.31)^ns^	1.18 (-0.05; 2.41)^ns^
Alcohol use	-1.02 (-1.71; -0.33)^**^	-0.63 (-1.34; 0.07)^ns^	-1.36 (-2.51; -0.21)^**^
Surgery	1.16 (0.11; 2.20)^*^	0.66 (-0.41; 1.74)^ns^	0.77 (-0.97; 2.51)^ns^
Chemotherapy	-0.17 (-1.05; 0.72)^ns^	-0.33 (-1.24; 0.58)^ns^	-0.05 (-1.52; 1.42)^ns^
Time			
Linear effect	0.39 (0.32; 0.47)^***^	0.74 (0.63; 0.84)^***^	0.43 (0.3; 0.56)^***^
Quadratic effect	-0.02 (-0.02; -0.02)^***^	-0.04 (-0.04; -0.03)^***^	-0.02 (-0.03; -0.02)^***^
Cubic effect	0.0002 (0.0002; 0.0003)^***^	0.0004 (0.0004; 0.0005)^***^	0.0003 (0.0002; 0.0004)^***^

*GC-RS*, general cancer-related symptoms; *HNC-RS*, head and neck cancer-related symptoms; *SIDA*, symptom interference with daily activities; *ECOG*, Eastern Cooperative Oncology Group; *HPV*, human papilloma virus; *CI*, confidence interval; *ns*, not significant; ^*^p < 0.05, ^**^p < 0.01, ^***^p < 0.001 (two-tailed)

As Fig. 1b-d shows, HPV-positive patients showed higher score, thus, worse QoL during treatment, whereas HPV-negative patients had worse QoL in the follow-up period, specifically when considering the HN cancer-related symptoms and the symptom interference with daily activities factors.

## Discussion

In this prospective longitudinal study, we used the PROM MDASI-HN to detect patients’ symptoms burden and implement interventions and therapy adjustments specific to each patient. A 3-factor solution, including GC-RS, HNC-RS, and SIDA, was considered, and a series of linear mixed model analyses were conducted. In both GC-RS and HNC-RS domains, time was the only significant predictor of patient’s QoL, whereas concerning the SIDA, time and HPV status were significant, resulting in HPV-positive patients with worst QoL than negative ones. It was evident that HNC patients’ QoL declined during RT (Fig. 1a), especially those symptoms specific to HNC, such as problems with mucus and difficulty in swallowing, that resulted to be more painful; nonetheless, QoL slowly improved as soon as treatment ended, which is consistent with the pattern found by other findings [28–32]. Indeed, it is plausible that symptom severity is worse during RT because of tumor presence as well as therapy short-term side effects, which consequently affect patients’ life, whereas after therapy completion, there should be a physical relief due to tumor size reduction, thus, an improvement of patients’ perception of their life quality.

However, it is also important to consider those findings in which side effects and problems persisted up to 1-year follow-up [31] and even beyond it [12, 28]. In these cases, the sequelae were related to specific HNC-related symptoms, such as dry mouth, sticky saliva, or senses dysfunctions, showing that although general and global QoL recovered, the same did not happen for specific HNC symptoms. For instance, Oskam and colleagues [12] found that QoL decrease related to HNC specific symptoms persisted up to a period between 8 to 11 years post-diagnosis. A possible explanation is that these problems and symptomatology are long-term side-effects of treatments, which appear only years after therapy, whereas other symptoms, such as nausea or pain, are caused by the presence of tumor or treatment administration [11]. Among the studies found, only a few [33–35] employed the M.D. Anderson Symptom Inventory Head and Neck module (MDASI-HN), 28-item version, which was used to assess symptoms severity during RT as well as in the follow up period. Most of previous research [28, 29, 31] used QoL measures that were longer than MDASI-HN, although measuring similar dimensions; thus, future research could use this questionnaire to address patients’ QoL and avoid extra burden to them.

The same abovementioned analyses were conducted among oropharynx cancer patients, distinguished by HPV positive and negative. Concerning HPV-negative patients, only the variable of time resulted to predict patients’ QoL. Among HPV-positive patients, time resulted to be significant in all the three factors. Regarding the GC-RS factor, being female, those patients who underwent surgery, those with low educational level, or patients that have never drunk alcohol had a worst QoL. Moreover, older patients were likely to have decreased QoL. It seems understandable that patients who had surgery may be debilitated, thus, having low QoL; similarly, patients with low educational level may engage in unhealthy behaviors and have less resources to cope with their disease. In relation to the HNC-RS factor, patients restricted in physically strenuous activity (ECOG 1) or with high educational level had a better QoL than fully active patients (ECOG 0) or those with a lower educational level. As for ECOG, our results appear to be contradictory at the first glance. We need to underline that a good performance status is generally classified as state 0 or 1one for the other. ECOG 0–1 is linked to better values in several scales of QOL. A possible explanation of our finding is that for patients with no functional impairment or premorbid lifestyle depicting a ECOG 0 status before starting RT, any impact on QOL is more perceived since the difference from baseline conditions is greater compared to patients with ECOG 1. For the SIDA, it was found that older patients, female subjects, those patients who were employed, or those who never used alcohol showed worst QoL. Unexpectedly, those subjects who never drink alcohol had worst QoL; this result would need to be further explored, considering that previous studies [36, 37] have focused on the prognostic role of alcohol use in developing HNC regardless its specific role during cancer treatment.

Comparing HPV-positive and HPV-negative patients’ QoL trends over time (Fig. 1b-d), it is possible to notice that although HPV-positive patients had worse QoL during treatment and immediately after it, especially in relation to GC-RS and HNC-RS factors, their QoL levels increase in the follow-up period; on the other hand, HPV-negative patients had worse QoL during the weeks after concluding treatment, thus, in the follow-up period. Our results are in agreement with literature data. Indeed, HPV-related oropharyngeal cancer patients’ population tends to be younger and healthier, with a very good baseline QOL, compared with individuals with other HPV-unrelated HNC. However, HPV-positive cancer patients are more likely to suffer a deterioration on their QOL during treatment. In a sub-study conducted within a prospective phase 3 randomized trial of concurrent standard radiation versus accelerated radiation plus cisplatin for locally advanced HN Carcinoma: NRG Oncology RTOG 0129, p16-positive oropharyngeal cancer (OPC) patients had better QOL than p16-negative patients did, before treatment and after 1 year after treatment. However, QOL/PS decreased more significantly from pretreatment to the last 2 weeks of treatment in the p16-positive group than in the p16-negative group [38]. Again, in a sub-analysis of the randomized trial Trans-Tasman Radiation Oncology Group (TROG) 02.02 (HeadSTART), HPV-positive patients showed a more dramatic QOL drop with concurrent chemoradiation compared to HPV-negative ones [39].

The current study has some limitations that should be noted and may have an influence on results generalization. First, due to drop-out the sample size of those who completed the questionnaire up to the last time point was smaller than the one who answered at the beginning of the research. Second, our sample consisted mainly of male patients with a prevalence of oropharynx tumors. Although the presence of these limitations, using the MDASI-HN, is a valid and short PROM, having a timeline that included both the treatment and the follow-up period resulted to be fundamental to have deeper understanding of patients’ QoL. Future research should give further attention to treatments sequelae specific to HNC, especially in the long-term period; extending the follow-up period would allow to better understand symptoms trajectories and their interference with daily life, considering that HNC specific symptoms may persist even years after ending treatments. Furthermore, it seems important to consider other psycho-social variables (for instance, gender and financial toxicity [40]), which may have an impact on treatment outcomes as well as patients’ QoL, and analyze their trajectories over time, allowing to understand how these variables interact with patients’ physical and psychological well-being. This would help to develop more specific treatments and interventions that would answer to patients’ needs.

## Conclusion

Although QoL is an important indicator of healthcare systems quality and is included within the assessment of treatments benefits [41], some of its aspects may be often underdiagnosed and thus undertreated by physicians [22, 42]. Moreover, clinical as well as socio-demographic variables may have an impact on patients’ QoL. Hence, PROM as a standard procedure should be included in patients’ condition assessment, allowing deeper insights of their disease experience and excluding response misunderstanding [35, 43, 44].

## Data Availability

Data are available from the authors upon reasonable request.

## Code availability

N/A.
